# Incidence and risk factors for cytomegalovirus in kidney transplant patients in Babol, northern Iran

**Published:** 2017

**Authors:** Arefeh Babazadeh, Mostafa Javanian, Farshid Oliaei, Roghayeh Akbari, Abazar Akbarzadepasha, Ali Bijani, Mahmoud Sadeghi

**Affiliations:** 1 Infectious Diseases and Tropical Medicine Research Center, Babol University of Medical Sciences, Babol, Iran.; 2 Kidney Transplantation Center, Shahid Beheshti Hospital, Babol University of Medical Sciences, Babol, Iran.; 3 Social Determinants of Health Research Center, Health Research Institute, Babol University of Medical Sciences, Babol, Iran.

**Keywords:** Cytomegalovirus, Incidence, Renal transplantation

## Abstract

**Background:**

Cytomegalovirus (CMV) disease is an important cause of death and possibly transplant rejection in kidney transplant (KT) patients. This study was conducted to investigate the incidence and risk factors of CMV disease in kidney transplant patients.

**Methods:**

All end-stage renal disease (ESRD) patients who underwent kidney transplantation during 1998-2014 and their donors were assessed. All samples were followed-up for approximately 70 months. CMV was identified by polymerase chain reaction (PCR) and/or PP65 antigen in peripheral blood leukocytes along with clinical manifestations.

**Results:**

A total of 1450 cases participated in the current study. CMV was diagnosed in 178 out of 725 (24.6%) kidney recipients. The annual incidence of CMV disease was 4.2%. Patients older than 40 years had a higher incidence of CMV disease. The level of CMV disease incidence in the 41-60 age group was 4 fold compared to those under 20 of age group (P=0.001).

**Conclusion:**

This study demonstrated that the incidence of CMV disease in our region is relatively low and also age more than 40 years and EBV infection are the important risk factors in kidney transplant patients. So care and monitoring of these patients are crucial in the first 5 months.

*Cytomegalovirus* (CMV) is one of the most important viruses of the Herpesviridae family infecting a large percentage of people worldwide. Seroprevalence of this virus varies in different regions ranging from 30 to 97 % (1). CMV disease is mostly reported in immune deficient individuals, such as kidney transplant recipients (2, 3, 4). It is considered a leading cause of morbidity and mortality in these patients; however, it is preventable (5). This infection typically occurs within the first three months after transplantation (6-8). In different studies, various risk factors have been reported such as environmental, geographical and economical. Other factors for CMV infection include host conditions like age, comorbidities, underlying disease, leukopenia, lymphopenia, cold ischemia time, genetic factors) along with overall immune status which is determined by the immunosuppressive protocol (type of medication, timing, duration of use). One of the most definitive risk factors expressed in various studies is the serological CMV incompatibility between transplant donor(D) and recipient(R), especially when the donor is positive and recipient is negative (9). 

Since there is very little data on CMV disease and its risk factors in Iran, particularly in the northern region, this study was performed to determine the incidence and risk factors of CMV disease in kidney transplant recipients in the North of Iran.

## Methods

**Population study: **Upon receiving the approval letter no. 30/5262 from the Ethics Committee of Babol University of Medical Sciences, this study was performed on kidney transplant recipients at Shahid Beheshti Hospital of Babol University of Medical Sciences which is the center for kidney transplantation in North of Iran with total population of over four million. 

Between the years of 1998 to 2014, 725 transplantation case files were stored in archives. Medical history, demographic data and follow-up information were gathered in a questionnaire. 

The questionnaire covers demographic data such as age, gender, weight, blood type, addiction and smoking for transplant donors and recipients. Medical conditions including hypertension, diabetes, ischemic heart disease (IHD), coronary artery bypass graft (CABG), dialysis type, immunosuppressive regimen, history of transplant rejection, history of blood transfusion and serological status of CMV and Epstein-Barr virus (EBV) in recipients were listed in the questionnaire and filled in using the hospital files. CMV was diagnosed using polymerase chain reaction (PCR) or pp65 antigen in peripheral blood leukocytes and the clinical manifestation was compatible with this disease. Exclusion criteria were any cases who received empirical therapy for clinical signs of CMV without confirmative test. 

Immunosuppressive regimens used for transplant patients were divided into three groups: 

1. cyclosporine and prednisolone

2. cyclosporine and mycophenolate mofetil (MMF)

3. prednisolone and different regimens containing thymoglobulin.

**Statistical analysis: **The collected data was analyzed using SPSS Version 22 software. Chi-square test and t-test were used to compare the qualitative and quantitative variables. Kaplan-Meier survival curve was used to analyze the possible risk factors. 

In addition, Cox's regression test used to evaluate patient survival hazard ratios with 95% confidence intervals was calculated. A p-value of less than 0.05 was considered statistically significant. 

## Results

Twenty patients who were not screened for CMV infection or were not completely followed-up were excluded from the study. Demographic data, blood type, addiction and smoking status of the patients are shown in table 1. 

**Table 1:** Demographic information of 725 kidney transplant recipients and donors.

**Variables**	**Recipients ** **N(%)**	**Donors** **N(%)**
Age(M±SD)	14.34±39.40	4.88±28.87
Weight(M±SD)	14.92±63.21	12.05±70.83
Gender (Male)	(60.8)441	(75.6)548
Addiction (+)	(2.2)16	(29.7)215
Smoking (+)	(9.7)70	(47)341
Blood type		
A B AB O	(25.9)188 (26.3)190 (8.4)61 (39.4)286	(23.0)167 (23.4)169 (4.8)35 (48.8)354
RH		
+ -	(92.0)667 (8.0)58	(90.1)653 (9.9)72

**Figure 1:**
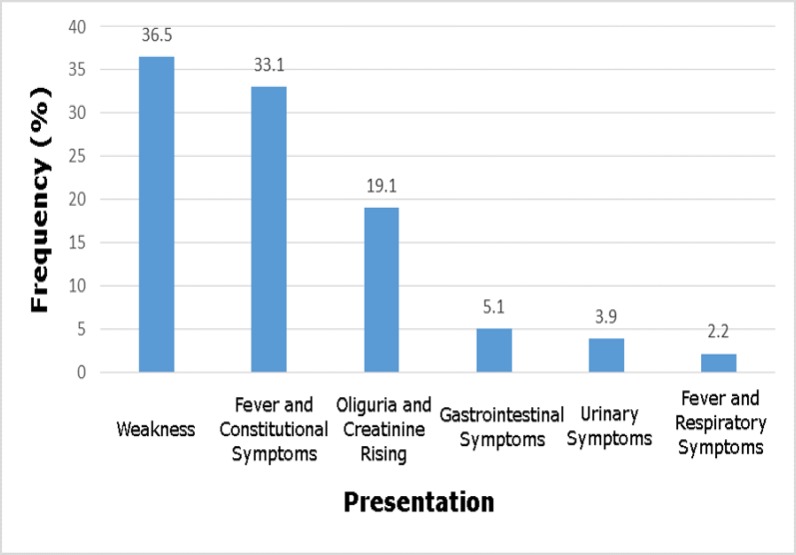
Frequency of some clinical manifestations of kidney transplant patients with cytomegalovirus disease in North of Iran.

**Figure 2:**
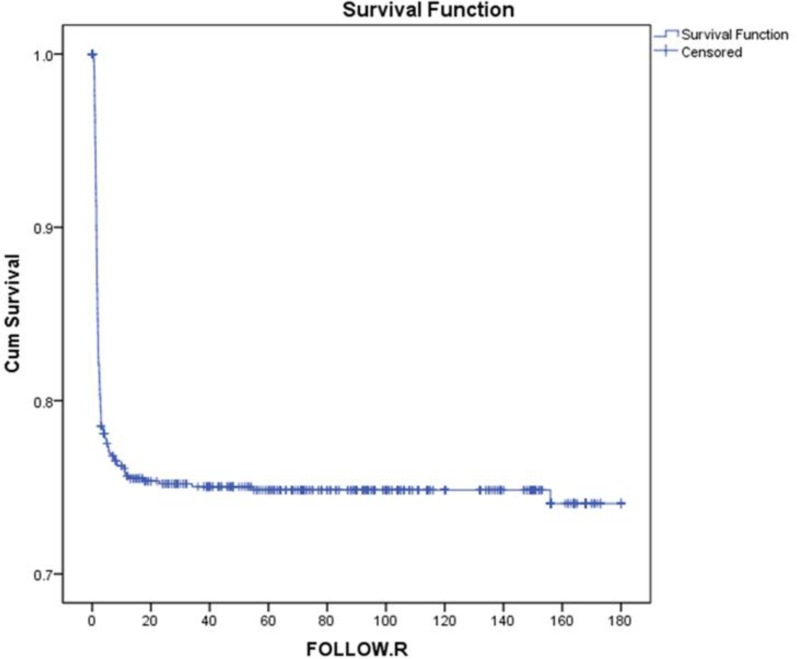
Incidence of cytomegalovirus disease in transplant patients in North of Iran using Kaplan-Meier method

The mean duration of follow-up was 69.81±10.59 month's equivalent to 4218 person/year. 720 out of 725 donors (99.3%) and 688 out of 725 (94.9%) recipients were seropositive for CMV infection. Most cases were donor (D) + / recipients (R) + and other serologic groups, including D - / R -, D + / R- and D- / R + were not considered. Out of the 725 recipients, 178 (24.6%) cases were diagnosed of CMV disease, therefore the incidence of the disease was 4.2% per year. The highest frequencies of CMV manifestations were weakness, fever and constitutional symptoms and the lowest were fever and respiratory symptoms, respectively (figure 1). CMV was diagnosed in 89.3% (159 out of 178) of transplant recipients during the first 5 months and the minimum reactivation time was 0.16 months (CI 95%:0.035-0.065) (figure 2). 

The data concerning cytomegalovirus disease and demographic data, immunosuppressive regimens, Epstein Barr Virus (EBV)-antibody are shown in table 2. Based on the Cox regression multivariate analysis, age of over 40 years and EBV seropositivity were considered as the major risk factors for of CMV disease in kidney transplant recipients (table 3).

**Table 2:** The percentage of cytomegalovirus disease and its risk factors in studied kidney transplant recipients of North of Iran

**Variables**	**CMV disease**		**P value**
	**Positive** **N(%)**	**Negative** **N(%)**	
Age group(year)			
0-20 21-40 41-60 61-80	(12.7)10 (19.6)56 (29.3)93 (44.2)19	(87.3)69 (80.4)230 (70.7)224 (55.8)24	0.0001>
Gender			
Male Female	(24.5)108 (24.6)70	(75.5)333 (75.4)214	0.9
Weight (Kg)			
0-60 60.1-120	(19.9)62 (28.0)116	(80.1)249 (72.0)298	0.01
Hypertension + _	(24.1)127 (25.8)51	(75.9)400 (74.2)147	0.69
Diabetes + _	(27.7)39 (23.8)139	(72.3)102 (76.2)445	0.38
IHD^1^ + _	(50.0)2 (24.4)176	(50.0)2 (75.6)545	0.25
CABG^2^ + _	(33.3)1 (24.5)177	(66.7)2 (75.5)545	0.99
Dialysis type			
No dialysis Hemodialysis Peritoneal dialysis	(16.1)10 (25.4)163 (22.7)5	(83.9)52 (74.6)478 (77.3)17	0.25
Immunosuppressive regimens			
Prednisolone + cyclosporine Cyclosporine + MMF^3^+ prednisolone Thymoglobulin containing regimen	(20.7)75 (29.8)85 (23.4)18	(79.3)288 (70.2)200 (76.6)59	0.02
History of transplant rejection + _	(7.7)1 (24.9)177	(92.3)12 (75.1)535	0.24
History of blood transfusion + _	(30.6)11 (24.2)167	(69.4)25 (75.8)522	0.42
Blood type			
A B AB O	(23.4)44 (28.4)54 (23.0)14 (23.1)66	(76.6)144 (71.6)136 (77.0)47 (76.9)220	0.55
RH			
+ -	(23.8)159 (32.8)19	(76.2)508 (67.2)39	0.15
EBV AB			
Not reported Seropositive Seronegative	- (25.6)172 (23.1)6	(100)27 (74.4)500 (76.9)20	0.005
CMV AB			
Not reported Seropositive Seronegative	- (25.4)175 (30.0)3	(100)27 (74.6)513 (70.0)7	0.28

1 . Ischemic heart disease

2 . Coronary artery bypass graft

3 . Mycophenolate-mofetil

**Table 3:** The risk factors of cytomegalovirus disease in kidney transplant patients of North of Iran based on Cox regression backward stepwise LR model

**Variables**	**Hazard ratio**	**Confidence interval 95%**	**P-value**
Age groups (Year)			
0-20 21-40 41-60 61-80	1 1.45 2.04 4.11	1 2.93-0.71 4.10-1.02 1.82-9.26	0.3 0.04 0.001
Weight (Kg)			
0-60 60.1-120	1 1.14	1 0.82-1.59	0.42
Immunosuppressive regimens			
Prednisolone + cyclosporine Cyclosporine + MMF^1^ + prednisolone Containing regimen thymoglobulin	1 1.29 0.92	1 0.89-1.85 0.50-1.70	0.29 0.16 0.80
Positive EBV AB	1.62	2.54-1.03	0.03

1. mycophenolate-mofetil

## Discussion

In this study, 99.7% of transplant donors and 94.9% of transplant recipients were seropositive in terms of CMV infection; these results are similar to the seroprevalence reported from developing countries such as Colombia and the Philippines (10-14) which reported the seroprevalence as over 90% among the transplant donors and recipients. Our results were much higher than the developed countries such as the USA (only 12% of donors and 19% of recipients) and the UK (29.1% of transplant recipients) (15, 16). This difference may be due to variations in cultural, economical, racial, social and health habits in these countries (17, 18, 19). The annual incidence of cytomegalovirus disease in our study was 4.2%, which is similar to the incidence rate reported in the Philippines (5.8%) and Colombia (7.25%) (11, 14). This may be due to the similarity of the socioeconomic and cultural circumstances of these countries, causing high CMV seroprevalence in the population. Therefore, the serologic compatibility status (D+ / R +) between transplant donor and recipient is common and the D+/R- status which is a major risk factor of CMV is not prevalent (9). 

In contrast, some studies also had a higher incidence than our study (14% to 38%) (15, 20-23). A possible reason for this difference is that unlike our study, other reports state that the serologic incompatibility of CMV was higher between donors and recipients. The current study showed that age higher than 40 was a major risk factor of CMV. Similarly, a study performed by Diaz showed that an increase of age was a risk factor for the incidence of CMV disease (p<0.001) (14). In a survey conducted by Nemati, cases aged higher than 55 years were 3.83 times more at risk for *Cytomegalovirus* (CI95%=1.21-12.14) (P=0.02) (24). Additionally, a study performed in the USA demonstrated that the prevalence of *Cytomegalovirus* was varied in different age groups ranging from 36.3 to 90.8% in the 6-11 and over 80 age groups, respectively (25). This increase of incidence may possibly be the result of a weakened immune system in old age. However, in some studies higher age was not a risk factor for CMV disease (11, 12, 20, 26, 27).

In this study, the type of immunosuppressive regimen did not influence the CMV incidence which was similar to the studies carried out by Bataille et al., 2010, Nafar et al., 2014 and Adu et al., 2007 (12, 26, 28). In contrast, some studies reported that treatment with MMF, OKT3 and thymoglobulin caused an increased risk for cytomegalovirus disease (14, 28, 29). 

This may be due to differences in sample size, serologic status and racial differences. Interestingly, the current study found that EBV seropositivity predisposes the renal transplant recipient to CMV disease. This finding is not reported in other studies, perhaps EBV immunosuppression induced the needs to be clarified (30-35)

In this study, comorbidities such as diabetes and hypertension were not risk factors for the incidence of CMV disease which was similar to other studies (14); nevertheless some reports stated that diabetes was a risk factor, which is most likely due to the distribution of different age groups (24, 36-38). This study found no relationships between the serology of donors, the duration of dialysis before transplantation, ESRD, gender, smoking, or addiction with the incidence of CMV disease (39). Regardiess, some studies stated gender and prolonged dialysis before transplantation as risk factors of CMV (14, 24, 40). 

This study demonstrated that the disease occurred within the first five months after transplantation which was inconsistent with several studies (11, 14, 41). The most common manifestations of CMV disease in the studied population were weakness, fever and constitutional symptoms, and respiratory symptoms were the rare manifestations. These findings are similar to other studies (11, 14). 

The limitations of our study were the unequal number of patients using immunosuppressive drugs, lack of viral load measures due to the use of qualitative PCR, lack of age categories and incomplete information**.**

In conclusion, the incidence of CMV disease in kidney transplant recipients in our region is relatively low (4.2%). Age higher than 40 and possible EBV seropositivity were the greatest risk factors for CMV disease, thus it seems that ESRD patients should undergo transplantation as quickly as possible. Contrary to what was expected, diabetes was not a risk factor for CMV disease and there were no differences between different immunosuppressive regimens. The first 5 months after transplantation are considered high risk for CMV disease, hence monitoring the patients more accurately is advisable,
